# Direct Rapid Left Ventricular Wire Pacing during Balloon Aortic Valvuloplasty

**DOI:** 10.3390/jcm9041017

**Published:** 2020-04-03

**Authors:** Pawel Kleczynski, Artur Dziewierz, Sylwia Socha, Tomasz Rakowski, Marzena Daniec, Barbara Zawislak, Saleh Arif, Joanna Wojtasik-Bakalarz, Dariusz Dudek, Lukasz Rzeszutko

**Affiliations:** 1 Department of Interventional Cardiology, Jagiellonian University Medical College, John Paul II Hospital, Pradnicka 80 Street, 31-202 Krakow, Poland; 2 2nd Department of Cardiology, Jagiellonian University Medical College, University Hospital, Jakubowskiego 2 Street, 30-688 Krakow, Poland; adziewierz@gmail.com (A.D.); mcrakows@cyfronet.pl (T.R.); Zawislak.barbara@gmail.com (B.Z.); mcdudek@cyfronet.pl (D.D.); 3 Department of Cardiology and Cardiovascular Interventions, University Hospital, Jakubowskiego 2 Street, 30-688 Krakow, Poland; sylwka.s27@gmail.com (S.S.); marzena.daniec@gmail.com (M.D.); saleharef@yahoo.gr (S.A.); j.wojtasik.bakalarz@gmail.com (J.W.-B.); lrzeszutko@cathlab.krakow.pl (L.R.); 4 Intensive Cardiac Care Unit, University Hospital, Jakubowskiego 2 Street, 30-688 Krakow, Poland

**Keywords:** aortic valve stenosis, balloon aortic valvuloplasty, wire, pacing

## Abstract

Background: Rapid ventricular pacing is mandatory for optimal balloon positioning during aortic valvuloplasty (BAV) in patients with severe aortic stenosis. We aimed to assess the safety and efficacy of direct left ventricular (LV) guidewire pacing in comparison with regular pacing induced by temporary pacemaker (PM) placement in the right ventricle. Methods: Direct rapid LV pacing was provided with a 0.035″ guidewire. Baseline clinical characteristics, echocardiographic and procedural data, as well as complication rates, were compared between the two groups. Results: A total of 202 patients undergoing BAV were enrolled (49.5% with direct LV guidewire pacing). The pacing success rate was 100%. In the direct LV guidewire pacing group, we found a lower radiation dose, shorter fluoroscopy and overall procedural time (0.16 vs. 0.28 Gy, *p* = 0.02; 5.4 vs. 10.3 min, *p* = 0.01; 17 vs. 25 min, *p* = 0.01; respectively). In addition, the complication rate was lower in that group (cardiac tamponades, vascular access site complications, blood transfusions rate, and in-hospital mortality: 0% vs. 3.9%; 4.0% vs. 15.7%; 2.0% vs. 12.7%; 2.0% vs. 9.8%, *p* = 0.01 for all, respectively). Conclusions: Direct rapid LV guidewire pacing is a simple, safe and effective option for BAV with a reduced complication rate compared to a temporary PM placed in the right ventricle.

## 1. Introduction

In a specific subset of patients with symptomatic aortic valve stenosis (AS), who are poor candidates for any definitive treatment due to severe comorbidities, balloon aortic valvuloplasty (BAV) can either serve as a standalone palliative procedure or as a bridge to final therapy [1]. Moreover, BAV allows patients with severe AS to undergo an urgent non-cardiac surgery with its good immediate result [2]. On the contrary to a transcatheter aortic valve replacement (TAVR), the long-term clinical and hemodynamic outcomes of BAV were shown to be rather poor [3,4,5,6]. When performing a BAV, rapid ventricular pacing is required to achieve a cardiac standstill during the balloon inflation at the aortic site. It is provided, in most cases, with a temporary pacemaker (PM) inserted into the right ventricle (RV), which may increase the risk of major complications, including cardiac tamponade. There is an ongoing need for the simplification of structural interventions heading towards a minimally invasive approach. That is why direct left ventricle (LV) guidewire pacing was introduced. However, the data regarding the direct pacing LV approach are scarce. Thus, we aimed to evaluate the pacing success rate and pacing-related complications of direct rapid LV guidewire pacing and pacing induced by a temporary PM placed in the RV in patients undergoing BAV.

## 2. Materials and Methods

We included the data of all consecutive patients with severe symptomatic AS with an aortic valve area (AVA) < 0.7 cm^2^ (indexed AVA< 0.5 cm^2^/m^2^ body surface area) who underwent BAV between January 2015 and May 2019 at our institution. LV pacing, however, was set as a standard approach in March 2018. All procedures before March 2018 were performed with RV pacing. The study was conducted as a single-center, prospective registry. Patients were clinically evaluated to assess the operative risk, comorbidities, frailty and procedural feasibility. Patient screening and selection were performed by a multidisciplinary ‘Heart Team’ supported by clinical and imaging resources. The study was approved by the institutional ethical board (122.6120.2.2017, 26 January 2017).

**Figure 1:**
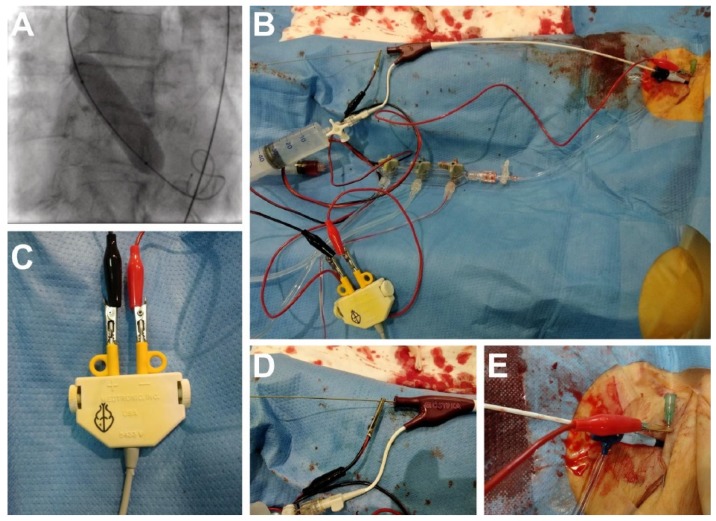
(**A**) Fluoroscopy of the rapid wire pacing during the balloon aortic valvuloplasty. (**B**) Whole set up overview: an alligator clamp connected to the 0.0035” guidewire in the left ventricle; the insulation guaranteed by the valvuloplasty balloon. (**C**) Both alligator clamps connected to the anode and cathode. (**D**) An alligator clamp connected to the 0.0035” guidewire. (**E**) A needle inserted into the groin connected with an electrode via an alligator clamp.

BAV was preceded by coronary angiography in most cases and percutaneous coronary intervention (PCI). BAV was guided by transthoracic echocardiography (TTE) and fluoroscopy and it was performed via puncture of the femoral artery, starting with a large sheath designed for coronary angiography/PCI. Unfractionated heparin was given to achieve an activated clotting time of 250 to 300 s. VACS II balloons from Osypka Medical Inc. (Berlin, Germany) were used in all cases. Different balloon sizes were used for each patient depending on a minimal annulus diameter measured in TTE. Exact positioning of the balloon was obtained by rapid ventricular pacing from either the 0.0035” ultra-stiff guidewire inserted into the LV or the regular endocavitary electrode placed in the RV (via a 6 or 7 Fr venous sheath). Direct LV pacing was obtained by connecting one electrode with an alligator clamp to the patient’s skin via a small needle inserted into the groin and one electrode connected through the alligator clamp with the LV guidewire (Figure 1). Insulation was ensured by the balloon catheter. In the case of primary LV pacing failure, additional partial wire braid removal with a scalpel provided successful pacing. The modification of the wire was performed in a proximal part just behind the shaft of the valvuloplasty balloon, where the alligator clamp was to be attached (Figure 1D). The number of balloon inflations was left to operator’s discretion. Vascular accesses were closed with manual compression or with an Angio-Seal (St. Jude Medical, Sint Paul, MN, USA) vascular closure device. Baseline clinical, echocardiographic and procedural data, as well as complication rates, were analyzed.

## 3. Results

A total of 202 patients undergoing BAV were enrolled. The group of direct LV guidewire pacing (LVP group) consisted of 100 patients, while a group of temporary PM pacing included 102 patients (RVP group). Primary direct wire LV pacing was successful in 95.0% of patients. In 5.0% of patients, additional partial wire braid removal with a scalpel provided successful pacing and overall pacing success reached 100%. There was no difference in the baseline clinical and echocardiographic data (Table 1) between the groups. A 6 Fr venous sheath was used in 91.1% (93 patients). Concomitant coronary angiography was performed in 96% of cases with subsequent PCI in 21.3% of cases. Patients were treated with dual antiplatelet therapy during the index procedure in 36.0% of cases in the LVP group and 33.3% in the RVP group (*p* = 0.63). In the LVP group radiation dose, fluoroscopy time and overall time of the procedure were reduced compared to the RVP group (0.16 vs. 0.28 Gy, *p* = 0.02; 5.4 vs. 10.3 min, *p* = 0.01; 17 vs. 25 min, *p* = 0.01; respectively), (Table 2). Cardiac tamponade occurred more often in the RVP group (0.0% vs. 3.9%, *p* = 0.01). In two cases, tamponade was successfully treated with pericardiocentesis and in two remaining patients urgent cardiac surgery was required. Cardiac tamponade was caused by the temporary PM placed in the RV in two cases confirmed by cardiac surgeons. In two other cases, tamponade was treated successfully with pericardiocentesis. At least one case was caused by the PM inserted into the RV, based on echo images. In one case, it was difficult to determine the cause of tamponade, however, no annulus rupture was noted. Vascular access site complications occurred less frequently in the LVP group (4.0% vs. 15.7%, *p* = 0.01)—Table 3. Pseudoaneurysms were arterial complications. Hematomas could be related both to the venous and arterial access as well as the arteriovenous fistula. The need for blood transfusion was more frequent in the RVP group (2.0% vs. 12.7%, *p* = 0.01). In-hospital mortality was lower in the LVP group (2.0% vs. 9.8%, *p* = 0.01). In the LVP group, two deaths occurred due to cardiogenic shock during or immediately after the “real” rescue BAV, following cardiac arrest (pulseless electrical activity (PEA)) with unsuccessful resuscitation. In the RVP group, there were three intraprocedural deaths in patients with cardiogenic shock undergoing rescue BAV, resulting from subsequent cardiac arrest (asystole or PEA). Additionally, there were two fatal cardiac tamponades related to the PM inserted into the RV (one of them confirmed by echo images), three patients died due to acute respiratory failure despite mechanical ventilation and two more had a sudden cardiac arrest (fatal ventricular tachycardia or fibrillation) in the intensive care unit on the third day after the index procedure. There was no need for the urgent implantation of a PM after BAV. We also found a trend towards a shorter hospital stay in the LVP group compared to the RVP group (median (IQR) 6.0 (6.0–10.0) days vs. 10.0 (7.0–12.0) days, *p* = 0.06). Two patients (2.0%) with hip endoprosthesis presented with acute pain in the groin during rapid LV pacing.

## 4. Discussion

Nowadays, BAV is experiencing its uprising due to the exponential increase in TAVRs in recent years but also as a standalone procedure in developing countries with a limited reimbursement of TAVR. Moreover, BAV alone may be considered as a bridge to aortic valve replacement (AVR) or TAVR in symptomatic patients with AS who are poor candidates for any definitive treatment due to severe comorbidities [1]. Additionally, BAV sometimes allows patients with severe AS to undergo an urgent non-cardiac surgery with its good immediate result [2,7]. In the current study, we evaluated a technical feature of the pacing method during BAV with direct LV pacing via guidewire. Furthermore, there is an ongoing need for the simplification of structural interventions heading towards a simplified and less invasive approach to avoid complications [8]. The major finding of this study is that the presented method is effective and allows for a reduced radiation dose, fluoroscopy time and overall procedure time and is associated with a lower complication rate. 

Traditionally, pacing for BAV before and during balloon inflation requires the use of a temporary pacing lead inserted into the RV. However, the insertion process may contribute to a higher vascular complication rate and longer overall procedural time. RV pacing via guidewire is also possible, but it again requires additional venous access [9]. Left ventricular direct wire pacing was first introduced in 2007 as a novel method to facilitate the easier performance of BAV, with no additional PM or programming of existing pacing systems [10]. LV direct pacing was performed on pediatric patients with congenital aortic stenosis, with noted reduced vascular complications, decreased incidence of aortic insufficiency and shorter fluoroscopy time [10]. In another study where adult patients were enrolled, the authors noted similar positive experiences, with pacing efficacy equal to that of standard RV pacing [11]. However, the use of the LV pacing technique ended due to the early TAVR protocols requiring a PM insertion into the RV to provide appropriate rapid pacing and protection in case of arrhythmias and heart blocks seen more frequently in early generation valves [12].

Luckily, more data have recently been published in terms of direct LV pacing but combined with TAVR protocols. In the study from Faurie et al., on 38 patients undergoing BAV alone and 87 patients undergoing TAVR, a reduction in procedural time, incidence of vascular complications, and cardiac tamponade was observed [13]. Successful stimulation was achieved in all cases. Patients undergoing BAV in that study had no tamponade and fewer vascular complications compared to the LVP cohort of our study. This difference might be related to a smaller sample size. However, in 2.6% of patients, new and permanent PM implantation was required, which did not occur in our study. In another study by Hilling-Smith et al., 76 patients undergoing BAV alone and 110 patients undergoing BAV with TAVR using only LV pacing were enrolled [14]. This study provided similar findings: consistent pacing, reduced procedural time, reduced vascular complications and a possibility of early patient mobilization. Similar results on direct LV pacing during TAVR were reported in two small studies [15,16]. Recently, the results of a prospective, multicenter, randomized controlled trial were published by Faurie et al. [17]. Patients undergoing transfemoral TAVR with a SAPIEN valve (Edwards Lifesciences, Irvine, CA, USA) were allocated to LV (*n* = 151) or RV (*n* = 151) stimulation. Compared to RV stimulation, LV stimulation during TAVR was associated with a reduced procedure duration, reduced fluoroscopy time, reduced cost as well as a similar efficacy and safety [17].

There is also a possibility of BAV without rapid pacing, which can be an even less invasive and better tolerated method with comparable acute outcomes [18,19]. Nonetheless, lack of any pacing may result in balloon instability during inflation unless using a dog-bone-shaped balloon requiring, however, a larger introductory sheath [20]. A study by Dall’Ara et al. comprised 51 patients in the BAV group in whom rapid pacing was performed and 49 patients in the BAV group without rapid pacing. Acute hemodynamic and 30-day clinical outcomes were comparable in both groups. However, fewer people in the no pacing group complained of poor tolerance to the procedure. Moreover, temporary PM implantation and the incidence of moderate/severe renal function worsening were less common in the no pacing group [18]. In a study by Witzke at al. with 111 consecutive patients undergoing BAV, the authors showed similar procedural safety for both techniques but less efficacy in terms of a smaller post-procedural AVA for the rapid pacing technique, despite easier balloon stabilization [19]. 

In our study, the complication rate was lower in the LVP group. However, it is difficult to determine whether deaths were directly related to complications in the RVP group. Two cases of cardiac tamponades were definitely linked to the temporary PM insertion into the right ventricle. Vascular complications were either arterial or venous. Hematomas at the puncture site could be related both to venous and arterial access as well as AV fistula and more punctures at access sites contribute to a higher risk of vascular complications.

Nonetheless, we should also mention several limitations or technical difficulties related to the direct LV wire pacing. The tip of the guidewire should be placed in the LV apex with strict contact with the LV endocardium. This may sometimes be challenging, especially in the case of a poorly fixed system with the wire moving inside the LV and detaching from the LV wall. To prevent this situation, the operator should push the guidewire towards the LV, which may increase the risk of cardiac tamponade. In our study, the final success rate of wire pacing was 100%, but in previous studies the authors reported successful pacing in 84.9%–100.0% of cases [13,14,17]. In the case of initially unsuccessful wire pacing, we scraped the distal portion of the wire where the alligator clamp should be attached with subsequently ensured pacing. 

## 5. Limitations

The current study was conducted as a single-center prospective registry and not as a randomized controlled trial. All procedures were performed by three experienced operators. The sample size is relatively low to assess clinical end points properly. We did not perform any cost analysis. 

## 6. Conclusions

Direct rapid LV guidewire pacing is a simple, safe and effective method during BAV, which allows for a shorter procedural time and a reduced complication rate compared to a temporary PM placed in the RV.

## Figures and Tables

**Table 1:** Baseline clinical and echocardiographic characteristics.

	All (*n* = 202)	Left Ventricle Pacing Group (*n* = 100)	Right Ventricle Pacing Group (*n* = 102)	*p*-Value
Age, median (IQR) (years)	85 (82.1–88.3)	85 (83.3–89.4)	85 (82.3–89.5)	0.73
Men, *n* (%)	99 (49.0)	51 (51.0)	49 (48.0)	0.23
Body mass index, median (IQR) (kg/m^2^)	25.1 (23.7–27.6)	24.9 (23.4–28.3)	24.6 (23.1–27.9)	0.54
Estimated glomerular filtration rate, median (IQR) (mL/min/1.73 m^2^)	57 (40.5–70.4)	56 (39.2–72.1)	58 (41.4–74.5)	0.41
NYHA class, *n* (%)				0.51
I+II	0	0	0	
III	131 (64.8)	64 (64.0)	67 (65.6)	
IV	71 (35.1)	36 (36.0)	35 (34.3)	
Arterial hypertension, *n* (%)	192 (95.0)	93 (93.0)	99 (97.0)	0.15
Diabetes mellitus, *n* (%)	100 (49.5)	47 (47.0)	53 (51.9)	0.54
Atrial fibrillation, *n* (%)	59 (29.2)	29 (29.0)	30 (29.4)	0.86
Previous myocardial infarction, *n* (%)	48 (23.7)	22 (22.0)	26 (25.4)	0.63
Previous percutaneous coronary intervention, *n* (%)	67 (33.1)	35 (35.0)	32 (31.3)	0.21
Previous coronary artery bypass grafting, *n* (%)	34 (16.8)	18 (18.0)	16 (15.6)	0.72
Chronic obstructive pulmonary disease, *n* (%)	30 (14.8)	14 (14.0)	16 (15.6)	0.53
Peripheral artery disease, *n* (%)	32 (15.8)	15 (15.0)	17 (16.6)	0.79
Stroke/transient ischemic attack, *n* (%)	22 (10.8)	10 (10.0)	12 (11.7)	0.81
Syncope, *n* (%)	24 (11.9)	13 (13.0)	11 (10.7)	0.12
Previous heart failure deterioration, *n* (%)	109 (53.9)	55 (55.0)	54 (2.9	0.23
Cardiogenic shock, *n* (%)	10 (4.9)	6 (6.0)	4 (3.9)	0.1
Pacemaker, *n* (%)	16 (7.9)	7 (7.0)	9 (8.8)	0.87
Logistic EuroSCORE II, median (IQR)	8.1 (5.9–14.5)	9.4 (6.9–15.0)	8.5 (5.8–13.9)	0.22
The Society of Thoracic Surgeons score, median (IQR)	10.6 (8.2–12.6)	11.1 (9.3–13.5)	10.8 (8.5–13.2)	0.69
Maximal transaortic gradient, median (IQR) (mmHg)	95.8 (83.6–107.8)	97 (82.5–106.3)	94 (89.3–102.6)	0.56
Mean transaortic gradient, median (IQR) (mmHg)	42.1 (40.1–50.3)	40 (39.5–52.5)	44 (41.2–49.70	0.34
Aortic valve area, median (IQR) (cm^2^)	0.52 (0.45–0.59)	0.51 (0.43–0.59)	0.53 (0.45–0.58)	0.45
Left ventricle ejection fraction, median (IQR) (%)	46 (41.5–55.5)	48 (42.0–57.5)	47 (44.5–55.0)	0.67
Right ventricular systolic pressure, median (IQR) (mm Hg)	51 (39.0–63.0)	48 (38.5–62.0)	48 (35.0–64.5)	0.59

IQR—interquartile range; NYHA—New York Heart Association functional class.

**Table 2:** Procedural data.

	All (*n* = 202)	Left Ventricle Pacing Group (*n* = 100)	Right Ventricle Pacing Group (*n* = 102)	*p*-Value
Concomitant coronary angiography, *n* (%)	194 (96.0)	100 (100.0)	94 (92.1)	0.15
Concomitant PCI, *n* (%)	43 (21.2)	22 (22.0)	21 (20.5)	0.46
Size of femoral arterial sheath, median (IQR) (Fr)	9 (8.0–10.0)	9 (8.0–10.0)	9 (8.0–10.0)	0.94
Size of femoral venous sheath, median (IQR) (Fr)	6 (6.0–7.0)	-	6 (6.0–7.0)	
Heparin dose, median (IQR) (units)	5500 (4000.0–6500.0)	6000 (4500.0–7500.0)	5500 (3700.0–7000.0)	0.17
Number of inflations, median (IQR)	1 (1–2)	1 (1–2)	1 (1–2)	0.92
Vascular closure device, *n* (%)	67 (33)	32 (32.0)	35 (34.3)	0.57
Manual compression after sheath(s) removal, *n* (%)	135 (67)	68 (68.0)	67 (65.6)	0.89
Balloon size, median (IQR) (mm)	22 (18–23.5)	22 (18.0–23.5)	22 (18.0–24.0)	0.73
Radiation dose (BAV alone), median (IQR) (Gy)	0.2 (0.1–0.3)	0.2 (0.1–0.2)	0.3 (0.2–0.4)	0.02
Contrast media load (BAV alone), median (IQR) (mL)	10 (5.0–20.0)	10 (5.0–15.0)	10 (5.0–20.0)	0.82
Fluoroscopy time (BAV alone), median (IQR) (min)	7.4 (5.5–14.2)	5.4 (5.5–10.2)	10.3 (6.5–15.4)	0.01
Duration of the procedure (BAV alone), median (IQR) (min)	21 (18.9–31.5)	17 (15.5–22.0)	25 (19.5–31.0)	0.01
Maximal transaortic gradient after BAV, median (IQR) (mmHg)	63 (48.0–74.5)	61 (45.5–72.0)	64 (48.0–75.5)	0.71
Mean transaortic gradient after BAV, median (IQR) (mmHg)	32 (22.5–35.0)	30 (23.5–35.0)	33 (20.5–36.0)	0.67
Aortic valve area after BAV, median (IQR) (cm^2^)	0.78 (0.67–0.87)	0.78 (0.69–0.89)	0.77 (0.68–0.89)	0.65
Left ventricle ejection fraction after BAV, median (IQR) (%)	49 (45.5–58.0)	48 (45.9–60.5)	50 (43.0–58.5)	0.78

BAV—balloon aortic valvuloplasty; IQR—interquartile range; PCI—percutaneous coronary intervention.

**Table 3:** Complications.

	All (*n* = 202)	Left Ventricle Pacing Group (*n* = 100)	Right Ventricle Pacing Group (*n* = 102)	*p*-Value
Severe aortic regurgitation after BAV, *n* (%)	5 (2.0)	3 (3.0)	2 (1.9)	0.13
Cardiac tamponade, *n* (%)	4 (2.0)	0 (0.0)	4 (3.9)	0.01
Cerebrovascular incident, *n* (%)	3 (1.5)	1 (1.0)	2 (1.9)	0.15
Vascular access site complications, *n* (%)	22 (10.9)	4 (4.0)	16 (15.7)	0.01
hematoma, *n* (%)	10 (5.0)	2 (2.0)	8 (7.8)	0.01
pseudoaneurysm, *n* (%)	8 (3.9)	2 (2.0)	6 (5.9)	0.02
arteriovenous fistula, *n* (%)	2 (1.0)	0 (0)	2 (1.9)	0.03
retroperitoneal bleeding, *n* (%)	4 (2.0)	2 (2.0)	2 (1.9)	0.97
Blood transfusion, *n* (%)	15 (7.4)	2 (2.0)	13 (12.7)	0.01
Creatinine level before BAV, median (IQR) (g/dL)	116 (93.0–137.0)	114 (90.5–129.0)	118 (102.5–154.0)	0.42
Creatinine level after BAV, median (IQR) (g/dL)	117 (97.0–140.0)	119 (90.0–137.5)	123 (105.0–159.0)	0.32
Hospital stay, median (IQR) (days)	7 (6.0–10.5)	6 (6.0–10.0)	10 (7.0–12.0)	0.06
Intraprocedural mortality, *n* (%)	3 (1.5)	0 (0.0)	3 (2.9)	0.09
In-hospital mortality, *n* (%)	12 (5.9)	2 (2.0)	10 (9.8)	0.01

BAV—balloon aortic valvuloplasty; IQR—interquartile range.
